# Phenotypic and Transcriptomic Lymphocytes Changes in Allograft Recipients After Intravenous Immunoglobulin Therapy in Kidney Transplant Recipients

**DOI:** 10.3389/fimmu.2020.00034

**Published:** 2020-01-24

**Authors:** Caroline Pilon, Jeremy Bigot, Cynthia Grondin, Allan Thiolat, Philippe Lang, José L. Cohen, Philippe Grimbert, Marie Matignon

**Affiliations:** ^1^APHP (Assistance Publique-Hôpitaux de Paris), Hôpital H. Mondor-A. Chenevier, Centre d'Investigation Clinique Biothérapie, Créteil, France; ^2^Université Paris-Est, UMR_S955, UPEC, Créteil, France; ^3^Inserm, U955, Equipe 21, Créteil, France; ^4^APHP (Assistance Publique-Hôpitaux de Paris), Hôpital H. Mondor-A. Chenevier, Nephrology and Transplantation Department, Créteil, France

**Keywords:** kidney transplantation, high-dose intravenous immunoglobulin, donor specific antibodies, lymphocytes phenotype, immunomodulation

## Abstract

High dose intravenous immunoglobulin (IVIG) are widely used after kidney transplantation and its biological effect on T and B cell phenotype in the context of maintenance immunosuppression was not documented yet. We designed a monocentric prospective cohort study of kidney allograft recipients with anti-HLA donor specific antibodies (DSA) without acute rejection on screening biopsies treated with prophylactic high-dose IVIG (2 g/kg) monthly for 2 months. Any previous treatment with Rituximab was an exclusion criterion. We performed an extensive analysis of phenotypic and transcriptomic T and B lymphocytes changes and serum cytokines after treatment (day 60). Twelve kidney transplant recipients who completed at least two courses of high-dose IVIG (2 g/kg) were included in a median time of 45 (12–132) months after transplant. Anti-HLA DSA characteristics were similar before and after treatment. At D60, PBMC population distribution was similar to the day before the first infusion. CD8^+^ CD45RA^+^ T cells and naïve B-cells (Bm2^+^) decreased (*P* = 0.03 and *P* = 0.012, respectively) whereas Bm1 (mature B-cells) increased (*P* = 0.004). RORγt serum mRNA transcription factor and CD3 serum mRNA increased 60 days after IVIG (*P* = 0.02 for both). Among the 25 cytokines tested, only IL-18 serum concentration significantly decreased at D60 (*P* = 0.03). In conclusion, high dose IVIG induced limited B cell and T cell phenotype modifications that could lead to anti-HLA DSA decrease. However, no clinical effect has been isolated and the real benefit of prophylactic use of IVIG after kidney transplantation merits to be questioned.

## Introduction

Intravenous immunoglobulin (IVIG) is the most highly used therapies for immunodeficiencies, autoimmune, and inflammatory diseases (1). Despite the wide utilization of IVIG, the mechanisms supporting their immunomodulation properties remain not fully elucidated and somewhat controversial. Low-doses IVIG (0.3 g/kg) in common variable immunodeficiency induces proliferation and immunoglobulin synthesis from B cells (2). In the context of autoimmune and inflammatory diseases, dose of IVIG were elevated (2 g/kg) and their mechanisms of action were described to depend on Fc and/or F(ab')2 fragments (3). IVIG inhibit the activation and function of various innate immune cells such as dendritic cells, monocytes, macrophages, neutrophils cells, and NK cells (3) and neutralize activated complement components (3). In addition, IVIG modulate B-cell functions with a significant reduction in serum levels of BAFF (4) and plasma cells. In turns, IVIG enhances and restores the functions of Treg cells, induces apoptosis of activated effector T lymphocytes and down regulates the production of inflammatory cytokines (3). In addition, IVIG inhibits activation of endothelial cells, expression of adhesion molecules and secretion of soluble mediators (5).

In the field of kidney allograft transplantation, a number of preventive treatments in sensitized patients before transplant have been reported including high-dose IVIG and/or anti-CD20 antibody and/or plasmapheresis and/or bortezomib and/or monoclonal antibody to C5 (6–12). In the only randomized double-blinded trial analyzing high-dose IVIG in sensitized kidney allograft recipients, IVIG group displayed lower panel reactive antibody (PRA), higher rate of deceased-donor transplants, shorter time to transplantation, and similar 2-year graft survival albeit at the expense of higher rate of rejection (8). In another randomized clinical trial, adding rituximab to high-dose IVIG induced a significant decrease in acute antibody mediated rejection (ABMR) and improved kidney allograft function after transplant compared to IVIG alone (12). A retrospective study analyzed the benefit of adding plasmapheresis and rituximab to high-dose IVIG and depicted less microvascular inflammation and histological changes of chronic ABMR in the more intensive treated group compared to a historical cohort of control patients (11). Finally, the accurate role of IVIG in such clinical context remains difficult to establish and analysis of their action on humoral response is difficult to define since IVIG are mostly used in combined therapy strategies. Moreover, in the context of kidney transplant recipients receiving maintenance immunosuppressive drugs, the biological effect of high-dose IVIG treatment on T and B cell phenotype was not evaluated yet. Here, we proposed to analyze *in vivo* phenotypic and transcriptomic lymphocytes changes in kidney allograft recipients treated monthly with prophylactic high-dose IVIG (2 g/kg) because of *de novo* anti-HLA DSA (*dn* DSA) or preexisted DSA.

## Patients and Methods

### Study Design and Patients

We designed a monocentric prospective cohort study of kidney allograft recipients with significant anti-HLA DSA (before transplant (presensitized) or *dn* DSA) without acute rejection on protocol kidney allograft biopsy. A part of the cohort was treated with prophylactic high-dose IVIG (2 g/kg) monthly during 2 months between January 2013 and January 2014 and none were treated before with Rituximab. Prophylactic treatment was decided because of significant anti-HLA DSA (before transplant (presensitized) or *dn* DSA). Protocol kidney allograft biopsies were performed in our center to follow kidney allograft recipients with DSA before transplantation and *dn* DSA as acute ABMR is significantly higher in those patients (13, 14). Demographic and clinical information were collected before and after kidney transplantation. Tolerance of IVIG treatment were collected. Glomerular filtration rate (eGFR) was estimated with MDRD formula (15). Acute rejections were biopsy-proven in all cases and classified according to updated Banff classification (16). Allograft loss was defined with eGFR < 15 ml/min/1.73 m^2^ or the need for dialysis. This study was reviewed and approved by the Paris-4 institutional review board (CPP-APHP_2021).

### HLA Typing and Anti-HLA Donor Specific Antibodies Identification

HLA type was determined using high resolution typing for all donors and recipients. Participants were typed for class I loci (A, B, and CW) and class II loci (DR, DQ, and DP). Serum samples were systematically collected before IVIG treatment and 1 month after the last course of high dose IVIG to evaluate HLA sensitization. All serum samples were assessed with Luminex assays to determine the specificity of HLA class I and II IgG donor specific antibodies (DSA) (One Lambda Inc, CA). A baseline mean fluorescence intensity (MFI) value > 500 was considered positive. DSA characteristics analyzed included the absolute number, the highest MFI (MFImax) and the sum of MFI (MFIsum).

### Human Cell Isolation and Flow Cytometry

Peripheral blood was obtained from patients before each high-dose IVIG infusion (day 0 and day 30) and 1 month after completion of the two courses (day 60). Peripheral blood mononuclear cells (PBMCs) were isolated with lymphocyte separation medium (Laboratoires Eurobio, Les Ulis, France) and resuspended in phosphate-buffered saline (PBS; Life Technologies; Thermo Fisher Scientific, Waltham, MA) with 3% fetal bovine serum (FBS; Gibco, Life Technologies; Thermo Fisher Scientific). PBMCs were stained with various mAb combinations for 20 min at 4°C in staining buffer (PBS with 3% FBS). The directly conjugated mAbs anti-CD19-V500 (clone HIB19), CD56-APC (clone B159), CD14-PE-Cy7 (clone M5E2), CD3-V450 (clone UCHT1), CD4-PE (clone RPA-T4), CD8-APC (clone RPA-T8), CD45RA-FITC (clone L48), CD45RO-PerCP (clone UCHL1), CD38-PE-Cy7 (clone HB7) were supplied by BD Biosciences (France), IgD-FITC (clone IADB6), CD27-PE (clone IA4CD27) by Beckman Coulter (France), and Foxp3-eF450 (clone PCH101) by eBioscience (Thermo Fisher Scientific). Data were processed using FlowJo soft-ware (FlowJo LLC, Ashland, OR).

The gating strategy of the different cells subsets is presented in Supplemental Figure 1.

### RNA Isolation, Preamplification, and Reverse Transcription–Quantitative Polymerase Chain Reaction

Expression levels of 13 genes were analyzed using quantitative polymerase chain reaction (qPCR). Messenger RNA (mRNA) was extracted from PBMCs lysate (day 0, day 30, and day 60) using the RNeasy MiniKit (Qiagen, Hilden, Germany), according to the manufacturer's instructions and quantified on a nanodrop spectrophotometer. Total RNA was then reverse transcribed to complementary DNA (cDNA) with reverse transcriptase (Thermo Scientific, Courtaboeuf, France). Real-time quantitative PCR was performed with 13 commercially available primers and probe sets (Applied Biosystems, Foster City, CA) (HPRT: Hs99999909_m1, CD19: Hs00174333_m1, CD32a: Hs00234969_m1, CD32b: Hs00269610_m1, BAFF-R: Hs00606874_g1, BAFF: Hs00198106_m1, RORγT: Hs01076122_m1, Tbet: Hs00203436_m1, GATA-3: Hs00231122_m1, CD3: Hs00174158_m1, TGFβ1: Hs00998133_m1, Fas: Hs00236330_m1, FasL: Hs00181225_m1, CD4: Hs01058407_m1). This mechanistically informative panel of 13 mRNAs was designed based on our single center experience and as informed from the literature (17–19). The 2^−ΔΔct^ method was used to calculate the abundance of mRNAs in the samples and relative to reference controls. All samples were tested in duplicate in 96-well plates with the 7900HT fast real-time PCR system (Applied Biosystems). HPRT1 was used as an endogenous control to normalize RNA amounts.

### Cytokine Detection in Serum

Serum were harvested after centrifugation of whole blood (collected without any additives) from each patient at day 0, 30 and 60, and stored at −80°C. Cytokines were quantified using the Cytokine 25-Plex human ProcartaPlex Panel 1B with Luminex-based technology as specified by manufacturer (Thermo Scientific, Courtaboeuf, France). The following cytokines were analyzed: GM-CSF; IFN-α; IFN-γ; IL-1 α; IL-1 β; IL-1RA; IL-2; IL-4; IL-5; IL-6; IL-7; IL-9; IL-10; IL-12 p70; IL-13; IL-15; IL-17A; IL-18; IL-21; IL-22; IL-23; IL-27; IL-31; TNF-α; TNF-β/LTA.

### Statistical Analysis

Each patient was his own control. Continuous variables were expressed in mean Standard Deviation (SD) or median Interquartile Range (IQR) as appropriate. Categorical variables were expressed in N (%). Statistical analyses were adapted to data distribution (Mann–Whitney test, and unpaired or paired *t*-test). A *P*-value below than 0.05 was considered to be significant. Statistical analyses were performed using GraphPad Prism software (GraphPad Software, La Jolla, CA).

## Results

### Patients Characteristics

A total of 21 patients were included in the study. Among those, 12 were treated with prophylactic high-dose IVIG and 9 were not. All patients from treatment group completed at least two courses of high-dose IVIG (2 g/kg). Reason of prophylactic IVIG treatment was anti-HLA DSA detection without acute ABMR lesions on protocol biopsy. Clinical characteristics and transplant courses are depicted in Table 1. DSA characteristics are presented in Table 2. Both groups were comparable (Supplemental Table 1).

**Table 1:** Patients and kidney transplant characteristics.

**Variables**	**IVIG patients**	**IVIG free patients**	***P* value**
	***N* = 12**	***N* = 9**	
**Demographic**			
Women, *N* (%)	6 (50)	2 (22)	0.36
Age at the time of transplant, years, mean (SD)	48 ± 13	55 ± 15	0.29
**Initial nephropathy**			
Genetic disease, *N* (%)	1 (8)	2 (22)	0.09
Glomerular disease, *N* (%)	8 (67)	3 (33)	
Diabetes—Hypertension, *N* (%)	0 (0)	3 (33)	
Others, *N* (%)	3 (25)	1 (12)	
**Donor**			
Deceased, *N* (%)	12 (100)	8 (89)	0.43
Age, years, mean (SD)	50 ± 18	52 ± 14	0.74
**Mismatch number, median (IQR)**			
*Class I*	2.5 (2–4)	2 (2,3)	0.17
*Class II*	2 (0–3)	2 (1–4)	0.79
Cold ischemia time, hour, mean (SD)	18 ± 4	19 ± 6	0.89
**Immunosuppressive treatment**			
Induction, *N* (%)	8 (67)	9 (100)	0.10
R-Il2 antibody	4 (50)	4 (44)	0.67
Thymoglobulin	4 (50)	5 (66)	
Calcineurin inhibitors, *N* (%)	12 (100)	9 (100)	
Mycophenolate mofetil, *N* (%)	12 (100)	9 (100)	1.00
Steroids, *N* (%)	12 (100)	9 (100)	
**End of follow-up**			
eGFR, ml/min/1.73 m2, median (IQR)	49 (37–67)	61 (34–70)	0.60
Proteinuria, g/day, median (IQR)	0.1 (0–0.1)	0.2 (0.1–0.3)	0.97
Allograft loss, *N* (%)	1 (8)	0 (0)	1.00
Patient survival, *N* (%)	12 (100)	8 (89)	1.00

**Table 2:** Anti-HLA donor specific antibodies (DSA) characteristics evolution in both groups.

	**IVIG patients**			**IVIG-free patients**		
**Variables**	**At the time of immunomodulatory treatment**	**At day 90 after treatment**	***p*-value**	**At the time of immunomodulatory treatment**	**At day 90 after treatment**	***p*-value**
Patients, *N*	12	12		9	9	
Delay from transplant, months, median (IQR)	45 (12–132)			53 (25–72)		0.98
**CLASS I**						
Number, median (IQR)	0.5 (0–1)	0.5 (0–1)	1.00	1 (0.5–2)	0.5 (0–1.5)	1.00
MFI max, median (IQR)	2,647 (1,590–7,177)	1,896 (1,363–4,886)	0.09	4,334 (2,254–12,119)	7,267 (1,605–8,693)	0.68
MFI sum, median (IQR)	2,646 (1,028–10,886)	2,228 (1,237–9,031)	0.19	6,295 (3,814–1,3351)	7,267 (3,202–10,866)	1.00
**CLASS II**						
Number, median (IQR)	1 (1,2)	0.25 (1–2.75)	0.75	0.5 (1,2)	0.5 (1–1.5)	1.00
MFI max, median (IQR)	1,735 (973–9,544)	1,888 (773–13,909)	1.00	5,880 (848–10,506)	6,499 (1,369–9,489)	0.22
MFI sum, median (IQR)	1,911 (1,516–12,610)	3,310 (1,641–14,694)	0.36	6,967 (848–11,010)	7,511 (1,369–10,706)	0.37

Regarding IVIG treated patients, median delay of treatment was 45 (12–132) months after kidney transplant. Among the *N* = 8 patients receiving induction therapy, *N* = 4 were treated with thymoglobulin and *N* = 4 with RIL-2 antibody. One patient was treated 1 month before IVIG. Three patients presented with one episode of acute T cell mediated rejection, 24, 51, and 67 months before high dose IVIG treatment. All of them received steroids. The one occurring 24 months before was also treated with thymoglobulin. Delay from acute rejection to IVIG treatment and immunophenotyping analysis seemed to be reasonable as it was always more than 1 year. None of them received Rituximab before high-dose IVIG infusions. Delay between thymoglobulin and IVIG treatment was more than 12 months in all but one patient. Screening biopsies at the time of anti-HLA DSA detection were available in all patients (Supplemental Table 2). Histological analysis was strictly normal in nine patients. Two biopsies presented with isolated peritubular capillaritis grade 1 with no C4d positive staining nor glomerulitis. Chronic lesions included transplant glomerulopathy (*N* = 1), grade III IFTA (*N* = 3), and grade II IFTA (*N* = 2). DSA characteristics at the time of first IVIG infusion and 60 days after were described in Table 2. No difference can be isolated between the DSA before and after treatment (as in IVIG-free group—Table 2). One patient developed an acute ABMR 12 months after IVIG treatment completion. Clinical tolerance of high-dose IVIG was good without any adverse events besides benign headache in *N* = 4 patients. None of them presented with acute kidney injury.

### PBMC Phenotype Evolution After High Dose IVIG

We first analyzed the 21 patients included treated or not. Results are shown in Table 3. No difference could be isolated neither in PBMC population at day 30 and day 60 compared to day 0 (T-cell, B-cell and NK-cell) nor in subtypes.

**Table 3:** PBMC populations distribution in patients treated or not with IVIG.

**Populations**	**D0 (%.[Q1–Q3]) *N* = 21**	**D30 (%. [Q1–Q3]) *N* = 12**	**D30-0 *p*-value**	**D60 (%.[Q1–Q3]) *N* = 10**	**D60-0 *p*-value**
Monocytes (CD14^+^)	19.7 [14.4–33.9]	22.0 [16.6–32.3]	0.73	18.9 [7.6–35.3]	0.42
B cells (CD19^+^)	5.9 [2.5–10.6]	6.6 [3.4–11.1]	0.90	7.8 [4.0–9.3]	0.98
Bm1	18.2 [10.9–34.1]	17.6 [9.3–22.2]	0.67	20.7 [13.6–28.8]	0.37
Bm2	34.4 [26.0–63.0]	43.1 [29.2–58.1]	0.69	37.9 [24.6–56.9]	0.85
Bm2'	0.9 [0.6–2.2]	1.8 [0.8–3.0]	0.77	0.8 [0.7–1.8]	0.48
Bm3+4	0.7 [0.4–0.9]	0.6 [0.4–0.9]	0.96	0.7 [0.6–0.8]	0.89
Bm5	18.7 [8.5–27.7]	18.1 [11.0–26.3]	0.84	18.3 [8.9–27.8]	0.79
eBm5	8.1 [6.4–14.1]	10.0 [8.4–18.2]	0.15	10.5 [8.4–11.6]	0.53
T cells (CD3^+^)	50.7[32.6–61.2]	48.5 [39.1–58.8]	0.90	47.4 [44.5–69.2]	0.85
CD4^+^	55.3 [45.8–65.3]	54.3 [48.9–66.7]	0.83	53.6 [40.0–59.9]	0.53
CD45RA	25.1 [16.1–43.1]	22.6 [20.2–41.2]	0.86	21.0 [13.4–37.7]	0.54
CD45RO	53.8 [38.9–61.5]	51.8 [34.8–84.3]	0.93	58.2 [43.8–68.2]	0.49
Treg	6.5 [3.8–11.1]	5.6 [4.1–13.5]	0.94	6.8 [5.1–10.1]	0.57
CD8^+^	33.6 [28.1–42.3]	31.2 [27.7–42.8]	0.85	39.3 [32.0–46.2]	0.35
CD45RA	59.7 [43–72.9]	56.8 [42.8–68.5]	0.95	54.8 [37–71.1]	0.76
CD45RO	10.8 [6.6–20.9]	14 [7.6–21]	0.88	12 [8.2–28]	0.66
NK cells (CD56^+^)	12.7 [7.6–18.5]	12 [7.8–21]	1.00	11.7 [9.1–21.7]	0.59
NKT cells (CD3^+^CD56^+^)	5.7 [1.5–16.5]	6.2 [2.1–9.8]	0.89	8.8 [2–15.4]	0.44

**Figure 1:**
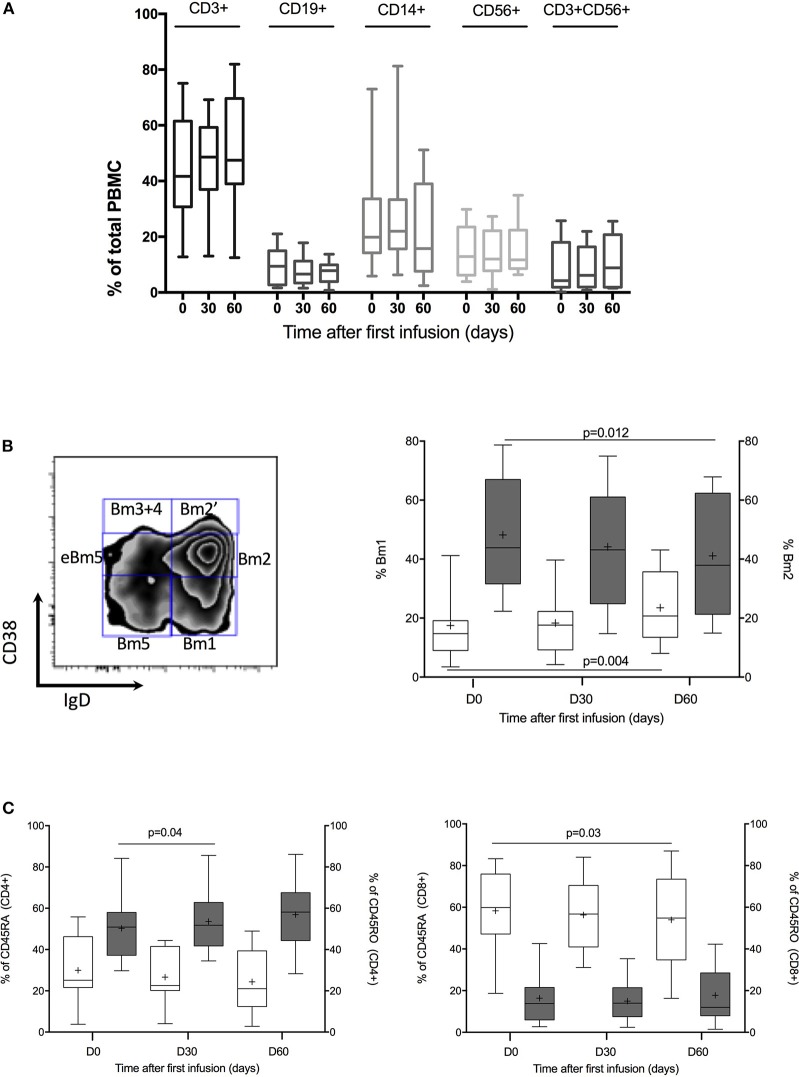
PBMC cells phenotypic analysis. **(A)** Kinetic of CD3^+^, CD19^+^, CD14^+^, CD56^+^, and CD3^+^CD56^+^ proportion throughout IVIG treatment. Box plot represent % of positive cells with 5–95% Whiskers. **(B)** B-cell population analysis. On the left, representative dot plots of B cells using the Bm1-5 classification based on CD38 and IgD expression. Gating strategies is shown. On the right % of Bm1 B cells (blanked) vs. % of Bm2 B cells (dark gray) at day 0, 30, and 60. Box plot represent % of positive cells with 5–95% Whiskers. Results were compared with Wilcoxon signed rank test. **(C)** CD45RA and CD45RO analysis in CD4 and CD8 cells. On the left, % of CD45RA (blanked) vs. % of CD45RO (dark gray) in CD4^+^ cells. On the right, % of CD45RA (blanked) vs. % of CD45RO (dark gray) in CD8^+^ cells. Box plot represent % of positive cells with 5–95% Whiskers. Results were compared with Wilcoxon signed rank test.

Then, as shown in Figure 1 and Table 4, we performed a matched analysis in the 12 treated patients and PBMC population distribution was similar whatever time before or after treatment and whatever the population considered: monocytes (CD14), B-cell lymphocytes (CD19), NK cells (CD56), NKT cells (CD3^+^CD56^+^) and/or T-cell lymphocytes (CD4 and CD8 among CD3). We next analyzed T-cell lymphocytes: CD45RA^+^ T cells (probably naive), CD45RO^+^ T cells (probably memory) and regulatory T cells (CD4^+^CD25^++^Foxp3^+^). Only CD8+ CD45RA^+^ cells decreased significantly at D60 (Figure 1C). We also analyzed T reg subtypes according to Miyara description (20). No changes could be isolated at day 30 and 60. We studied B-cell lymphocytes using Bm1-5 classification (Figure 1B and Table 3) (21). Two subgroups of B-cells were modified along the treatment. While Bm1 (mature B-cells) increased significantly (*P* = 0.004), Bm2 (naïve B-cells) decreased significantly (*P* = 0.012). No significant changes were observed on the other B-cells subsets. Next, we performed a discriminative analysis based on the induction therapy in the treated patients. According to small number of patients in each group (RIL2 antibody-group 2, thymoglobulin—group 1 and no induction—group 0), we analyzed variation considering each patient as his own control. No significant results could be highlighted excepted increase of Bm1 proportion in no induction treatment group at day 60 after treatment (Supplemental Table 2).

**Table 4:** PBMC populations distribution, serum cytokines and PBMC mRNA transcripts evolutions after IVIG treatment.

**Populations**	**D0 (%.[Q1–Q3]) *N* = 12**	**D30 (%. [Q1–Q3]) *N* = 12**	**D30-0 *p*-value**	**D60 (%.[Q1–Q3]) *N* = 10**	**D60-0 *p*-value**
Monocytes (CD14^+^)	19.9 [14.4–32.9]	22.0 [16.6–32.3]	0.79	18.9 [7.6–35.3]	0.16
B cells (CD19^+^)	9.4 [3.0–14.2]	6.6 [3.4–11.1]	0.15	7.8 [4.0–9.3]	0.11
Bm1	14.8 [9.9–19.0]	17.6 [9.3–22.2]	0.73	20.7 [13.6–28.8]	0.004
Bm2	43.8 [32.6–65.4]	43.1 [29.2–58.1]	0.15	37.9 [24.6–56.9]	0.01
Bm2'	1.1 [0.7–3.2]	1.8 [0.8–3.0]	1.00	0.8 [0.7–1.8]	0.64
Bm3+4	0.6 [0.3–0.8]	0.6 [0.4–0.9]	0.20	0.7 [0.6–0.8]	0.03
Bm5	16.7 [8.5–22.7]	18.1 [11.0–26.3]	0.18	18.3 [8.9–27.8]	0.09
eBm5	9.3 [7.1–14.1]	10.0 [8.4–18.2]	0.02	10.5 [8.4–11.6]	0.65
IgD+CD27- (naives)	60.7 [42.1–74.3]	56.4 [41.3–73.4]	0.46	55.8 [42.1–69.3]	0.34
Unswitched	6.8 [3.8–9.3]	9.6 [3.7–11.8]	0.46	7.6 [3.5–18.2]	0.31
IgD^+^CD27^+^ Switched IgD-CD27^+^	13.3 [8.5–31.7]	15.5 [9.7–28.6]	0.41	16.5 [10.6–32.2]	0.25
T cells (CD3^+^)	41.7[31.4–61.2]	48.5 [39.1–58.8]	0.73	47.4 [44.5–69.2]	0.06
CD4^+^	55.5 [46.2–63.6]	54.3 [48.9–66.7]	1.00	53.6 [40.0–59.9]	0.24
CD45RA	25.2 [21.6–43.7]	22.6 [20.2–41.2]	0.56	21.0 [13.4–37.7]	0.23
CD45RO	50.9 [38.9–57.4]	51.8 [34.8–84.3]	0.04	58.2 [43.8–68.2]	0.08
Treg	7 [3.6–15.8]	5.6 [4.1–13.5]	1.00	6.8 [5.1–10.1]	0.91
CD45RA^+^ Foxp3^+^ (I)	0.7 [0.4–1.7]	0.8 [0.6–1.5]	0.42	0.6 [0.6–1.0]	0.82
CD45RA- Foxp3^hi^ (II)	1.2 [0.2–2.0]	1.7 [0.8–2.3]	0.42	1.3 [0.7–1.7]	0.05
CD45RA- Foxp3^lo^ (III)	4.8 [2.2–13.1]	4.2 [3.5–7.8]	1.00	4.6 [4.1–5.9]	0.25
CD8^+^	32.6 [29.1–38.3]	31.2 [27.7–42.8]	0.92	39.3 [32.0–46.2]	0.24
CD45RA	59.9 [47.5–75.1]	56.8 [42.8–68.5]	0.63	54.8 [37–71.1]	0.03
CD45RO	13.9 [6–20.9]	14 [7.6–21]	0.70	12 [8.2–28]	0.43
NK cells (CD56^+^)	12.9 [7.6–22.4]	12 [7.8–21]	0.79	11.7 [9.1–21.7]	0.43
NKT cells (CD3^+^CD56^+^)	4.2 [1.9–15.7]	6.2 [2.1–9.8]	0.73	8.8 [2–15.4]	0.77
**Serum cytokines**	**D0 (pg/ml. [Q1–Q3])** ***N*** **=** **11**	**D30 (pg/mL. [Q1–Q3])** ***N*** **=** **11**	**D30-0** ***p*****-value**	**D60 (pg/mL. [Q1–Q3])** ***N*** **=** **8**	**D60-0** ***p*****-value**
IFNγ	6.85 [0.86–9.21]	6.17 [0–12.1]	1.00	9.88 [5.92–12.5]	0.38
IL-1RA	172 [16.9–524]	159.1 [65.5–240]	1.00	11.33 [0–343.8]	0.16
IL-7	7.55 [0.69–12.4]	7.49 [3.7–13.3]	0.94	7.54 [1.1–12.25]	0.64
IL-18	1.46 [0–9.97]	0 [0–3.68]	0.06	0 [0–5.6]	0.03
TNFα	0 [0–0.69]	0 [0–0.93]	0.50	0.47 [0–1.52]	0.07
**Genes**	**D0 (Fold increase. [Q1–Q3])** ***N*** **=** **11**	**D30 (Fold increase. [Q1–Q3])** ***N*** **=** **11**	**D30-0** ***p*****-value**	**D60 (Fold increase. [Q1–Q3])** ***N*** **=** **8**	**D60-0** ***p*****-value**
RORγT	1.00	1.5 [0.3–3.7]	0.29	2.0 [1.4–4.5]	0.02
Tbet	1.00	1.0 [0.5–1.6]	0.83	1.0 [0.6–3.6]	0.36
Gata-3	1.00	0.5 [0.3–1.0]	0.12	1.1 [0.3–2.0]	0.70
CD3	1.00	1.2 [0.8–2.6]	0.21	2.2 [0.8–3.2]	0.02
CD32a	1.00	1.0 [0.4–2.2]	0.90	0.9 [0.4–1.9]	1.00
CD32b	1.00	0.9 [0.3–2.5]	0.52	1.2 [0.6–2.3]	0.28
CD19	1.00	0.8 [0.6–1.5]	0.90	1.7 [0.7–2.9]	0.10
BAFF	1.00	1.3 [0.9–2.5]	0.06	1.6 [0.6–3.1]	0.10
BAFF-R	1.00	1.2 [0.6–3.0]	0.36	1.6 [0.8–2.0]	0.28
TGFβ	1.00	2.0 [0.6–3.0]	0.06	0.6 [0.4–1.6]	0.95
Fas	1.00	0.7 [0.3–3.3]	1.00	0.7 [0.2–1.4]	0.43
FasL	1.00	0.9 [0.3–1.2]	0.58	2.0 [0.6–4.6]	0.12
CD4	1.00	1.3 [0.4–2.2]	0.29	1.3 [0.9–1.8]	0.09

### PBMC mRNA Quantification Changes After High Dose IVIG

Transcript levels of molecules involved in inhibitory B-cell profiles (CD32 isoforms and the B-cell scaffold protein ankyrin repeats 1 BANK1), in B-cell survival (the B-cell activating factor BAFF and its receptor: BAFF-R), in T-cell differentiation (Th1, Th2, and Th17) and in apoptosis in the PBMC were analyzed in the treated patients. We justified the choice of targeted genes in Supplemental Data. Data were analyzed before treatment, at day 30 and at day 60 after two high dose IVIG infusions (Table 3). Levels of RORγt mRNA transcription factor and CD3 mRNA increased significantly 60 days after IVIG treatment beginning (*P* = 0.02 and *P* = 0.02, respectively). Correlation coefficient between CD3 transcriptomic and proteomic/cellular analysis was not significant (Supplemental Figure 2).

Discriminative analysis according to induction therapy did not reveal significant difference at day 30 and 60 compared to baseline (day 0) (Supplemental Table 3).

### Circulating Serum Cytokines Along High Dose IVIG Treatment

**Figure 2:**
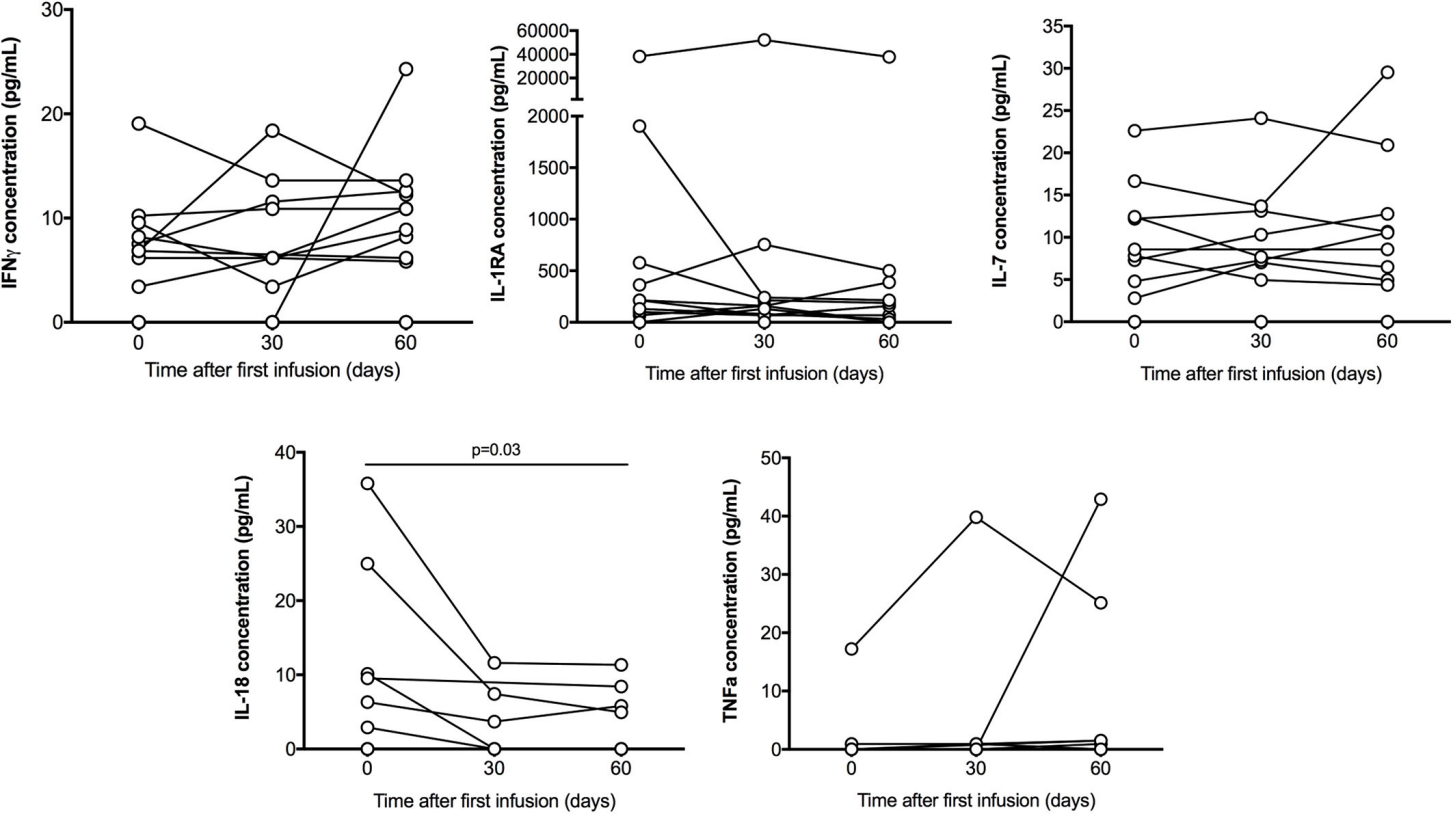
Serum cytokines multiplex analysis. IFNγ, IL-1R1A, IL-18, IL-7, and TNFα concentration (pg/mL) detected in patient throughout the treatment, on day 0, 30, and 60. Only IL-18 serum concentration significantly decreased at D60 (*P* = 0.03). Results were compared with Wilcoxon signed rank test.

Among the 25 cytokines analyzed in patients serum treated with IVIG, 11 where undetectable (IL-1α, IL-2, IL-5, IL-9, IL-10, IL-12p70, IL-13, IL-23, IL-31, IFNα, TNFβ), 9 where sporadically detected by one or two patients (IL-1β, IL-4, IL-6, IL-15, IL-17A, IL-21, IL-22, IL-27) and 5 were detected in more than 5 patients and are shown in Figure 2 and Table 3 (IL-7, IL-1Rα, IL-18, IFNγ, TNFα). Only IL-18 serum concentration significantly decreased at D60 (*P* = 0.03).

Considering discriminative analysis according to induction therapy, we could not analyze cytokines in each group due to small number of positive patients.

## Discussion

Prophylactic high dose IVIG has been used at the time of transplantation in high immunological risk recipients but analysis of their true action on humoral response is difficult to define since IVIG is mostly used as combined therapy (8, 22). In the context of *de novo* DSA in kidney allograft recipients, prophylactic use of high dose IVIG did not prevent acute ABMR and had minimal effects on DSA outcome (17). We provided here an extensive *in vivo* phenotypic and transcriptomic analysis in 12 patients treated with prophylactic high dose IVIG after kidney transplantation in patients with anti-HLA DSA without acute rejection and therefore without any other treatment associated. So more, the absence of acute rejection means the absence of immunological mechanisms activation or subclinical which will not interfere with IVIG actions.

Considering B lymphocytes, we showed first that proportion of naïve B-cells (Bm2) decreased significantly after high dose IVIG. This population was associated before and after transplant with anti-HLA DSA development within the first year after transplant (23, 24). In addition, belimumab, an anti-B lymphocyte stimulator (BLyS) antibody, associated at the time of transplant with standard transplant immunosuppression, decreased significantly naïve B-cells 24 weeks after transplant and *de-novo* IgG antibody formation (25). High dose IVIG could via the decrease of naïve B-cells (Bm2) limits *de-novo* IgG DSA formation. However, in our study, no early effect on DSA MFI has been reported. A later analysis merits to be performed in the future to look for a sustained effect on naïve B-cells and DSA.

In our study, high dose IVIG increased significantly, the proportion of memory B cells. Lower proportion of memory B cell before transplant, has been associated with high immunization levels (24) while higher proportion of memory B cell after transplant, has been described in operationally tolerant patients (no DSA and no immunosuppressive treatment) (26). In addition, in patients treated with belimumab proportion of memory B cell increased throughout the treatment with lower *de-novo* DSA (25). Whether increasing memory B-cell could decrease anti-HLA DSA production in our patients with immunomodulatory effects should be analyzed further.

High dose IVIG on T cell highlighted the decrease of the proportion of CD8^+^ CD45RA^+^ T cell after two infusions suggesting decrease of naïve T cell. Decreased frequency of CD8^+^ CD45RA^+^ and CD4^+^ CD45RA^+^ T cells have never been associated with acute rejection but with infection in older kidney recipients (> 60 years) (27). Increasing naïve T cells after transplantation could decrease memory T cells, interactions between T and B cells and may prevent not only alloantibody formation but also generation of long-lived memory T cells improving allograft survival. It is now well established that CD4^+^ and CD8^+^ memory T cells could contribute to allograft rejection and pose a major barrier to tolerance induction (28, 29).

RORγt mRNA transcripts, one nuclear factor involved in Th17 cells generation (30), increased significantly after high dose IVIG but without any changes in Th17 pathway cytokines. However, we did not analyzed RORγt using flow cytometry. Th17 cells and their cytokines, IL-17, IL-22, IFNγ, IL-6 and TNF-α, play a significant role in the development of acute and chronic allograft injury after organ transplantation (31). CD3 mRNA transcripts increased significantly while CD3 cells proportion remained stable. Only five cytokines could be analyzed because of low expression of cytokines in our patients. IL-18, which is a potent pro-inflammatory cytokine involved in the host defense by upregulating both innate and acquired immune responses especially Th1 responses (32), decreased significantly after IVIG high dose infusion. The main role of IL-18 is to crucially stimulate lymphocytes to produce the IFN-γ and regulate macrophages activity (32). However, in our study, serum levels decrease of IL-18 was not followed by a significant effect on serum levels of IFN-γ. Conclusion about transcripts levels modifications and cytokines expression after high dose IVIG could not be formulated.

Recently, Yabu et al. showed that immune profiles could predict response to desensitization therapy in highly HLA-sensitized kidney transplant candidates (33). More precisely, authors depicted a combination of transitional B cell and regulatory T cell frequencies before initiation of desensitization therapy that could distinguish responders from non-responders. The response to therapy was assessed by a predefined decrease of 5% or greater in cumulative calculated panel reactive antibodies. So more, one transcript, TRAF3IP3 could also distinguish responders from non-responders. However, we could not isolate responders from non-responders in our cohort because of the small number of patients, the absence of significant changes in DSA and only one acute ABMR episode after treatment.

The major limit of our study was the small number of patients included. However, after kidney allograft transplantation, high dose IVIG are never infused alone. Indications after kidney transplantation include (i) the prevention of acute ABMR in presensitized patients associated with thymoglobulin induction therapy and plasmapheresis and/or rituximab (ii) treatment of acute ABMR associated with plasmapheresis (11). In the first indication, no study evaluating prophylactic treatment in kidney deceased donor recipients is available. So more, prophylactic high doses IVIG are not used in clinical practice in patients with *dn* DSA and we stopped our pilot study because of failure to prevent acute ABMR (17). Considering clinical data without a net benefit and the cost of high dose IVIG, ~$6,500 for a single 140 g IVIG treatment (34), we could not treat more patients and our data *in vivo* could not be expanded. Meanwhile, we would like to highlight that this is the sole study analyzing high dose IVIG *in vivo* effect after kidney transplantation.

Taken together, our data (clinical and experimental) suggested that the benefits of high dose IVIG after kidney transplantation are limited. Furthermore, high dose IVIG have been associated with more tubular macrovacuoles and chronic tubulointerstitial changes after kidney transplantation (35, 36). Our results remain to be confirmed in a larger population which could lead to deep modifications of the current use of high dose IVIG in clinical practice after kidney transplantation.

In conclusion, we present here the first study analyzing *in vivo* prophylactic high dose IVIG effects on T and B cell phenotype in kidney allograft recipients with *de novo* DSA. Our results trend to suggest that high dose IVIG induce limited modifications in B and T cell phenotype including decrease of naïve B cell and CD8^+^ T cell and increase of memory B cell. These modifications could lead to immunomodulation and limit anti-HLA DSA production. However, our previously clinical data suggested that kidney allograft recipients with DSA treated preventively with high dose IVIG showed similar AMR rate than non-treated group (17) and no DSA modifications could be isolated in this study.

## Data Availability Statement

The datasets generated for this study are available on request to the corresponding author.

## Ethics Statement

The studies involving human participants were reviewed and approved by APHP-Saint Louis. The patients/participants provided their written informed consent to participate in this study.

## Author Contributions

CP, JB, CG, PG, and MM: conceptualization, methodology, and data curation. CP: software. CP, JB, CG, PL, JC, AT, PG, and MM: validation and formal analysis. MM: resources and supervision. CP and MM: writing—original draft preparation. CP, JB, AT, JC, PG, and MM: writing—review and editing.

### Conflict of Interest

The authors declare that the research was conducted in the absence of any commercial or financial relationships that could be construed as a potential conflict of interest.
