# Supplementary material for: Phenotypic and Transcriptomic Lymphocytes Changes in Allograft Recipients After Intravenous Immunoglobulin Therapy in Kidney Transplant Recipients
Source: Front Immunol. 2020 Jan 24;11:34. doi: 10.3389/fimmu.2020.00034 (PMC6993066; doi:10.3389/fimmu.2020.00034)
Supplement: Supplementary file 1 [file Data_Sheet_1.docx]

**Supplementary data**

1. **Targeted genes used justification**

***HPRT***: reference gene to normalize the amount of transcript.

***TGFb1***

TGF-β1 plays an important role in controlling the immune system, and shows different activities on different types of cell, or cells at different developmental stages. Most immune cells (or leukocytes) secrete TGF-β1.

Letterio JJ, Roberts AB (1998). "Regulation of immune responses by TGF-beta". Annu. Rev. Immunol. 16: 137–61.

***CD19***

B lymphocytes marker except for plasma cell and in follicular dendritic cells.

*Reference: Tedder et al. Journal of Immunology. 143 (2):712.*

***CD32a – CD32b***

CD32a and CD32b are surface receptor proteins acting to modulate signaling. Balance between both proteins lead to activation (CD32a) or inhibition (CD32b) of B-cell producing antibodies. We showed previously that IVIG treatment significantly decreased CD32a mRNA expression in circulating leucocytes.

*Reference: Veri et al. Immunology. 121 (3):392-404. Matignon et al. PLoS One 2017. 12 (6) :e0178572.*

***BAFF – BAFF-R***

B-cell activating factor and B-cell activating factor receptor. BAFF is a cytokine known to activate B-cell proliferation and differentiation. It has been shown that these two cytokines could be involved in tolerance and immunomodulation. BAFF-R signaling was demonstrated to determine transitional B-cell maturation and the subsequent survival of mature B cells. high levels of BAFF with abnormal renal function and HLA immunization or a local humoral alloimmune response in kidney transplantation.

*References: Kreuzaler et al. Journal of Immunology. 188 (1) :497-503. Tussiwand R et al European journal of immunology 2012;42(1):206-216.* *Thibault-Espitia A et al. American journal of transplantation: 2012;12(10):2754-2762.*

***Tbet – GATA-3 - RORgT***

T cell activation transcription factor. T-bet is largely expressed in case of Th1 differentiation, GATA-2 in Th2 differentiation and RORgT in Th17.

*Reference: Ye et al. Digestive diseases 28 (1):31-44 2010.*

***Fas - FasL***

Fas and its ligand are proteins implicated in programmed cell death (apoptosis). IVIG could block Fas-FasL interactions. This pathway could be involved in acute rejection.

*Reference: Krammer et al. Nature. 407 (6805): 789-95. Viard et al. Science 1998 282: 490-493.*

***CD4***

T lymphocytes marker. IVIG could suppressed CD4 T cell counts.

*Reference: Paquin-Proulx et al. PLos One 2013 ; 8(10) : e75199.*

***CD3***

T lymphocytes marker.

*Reference: Zheng et al. Nature. 573 (7775): 546-552.*

1. **Supplementary figures**

Supplementary figure 1 : gating strategy for phenotypic flow cytometry analysis.

Supplementary Figure 2 : correlation between CD3 mRNA transcripts and CD3 phenotype analysis. No correlation was found between both. Analyzes were performed using linear regression test.

1. **Supplementary tables**

Supplementary Table 1 : DSA characteristics at day 0 in both groups

| **Variables** | **IVIG patients** | **IVIG-free patients** | **P-value** |
| --- | --- | --- | --- |
|  |  |  |  |
| Patients, N | 12 | 9 |  |
| Delay from transplant, months, median (IQR) | 45 (12-132) | 53 (25-72) | 0.98 |
| Class I |  |  |  |
| Number, median (IQR) | 0.5 (0-1) | 1 (0.5-2) | 0.28 |
| MFI max, median (IQR) | 2647 (1590-7177) | 5631 (2535-12674) | 0.24 |
| MFI sum, median (IQR) | 2646 (1028-10886) | 6295 (3814-13351) | 0.31 |
| Class II |  |  |  |
| Number, median (IQR) | 1 (1-2) | 1 (0.5-2) | 0.76 |
| MFI max, median (IQR) | 1735 (973-9544) | 5880 (848-10506) | 0.81 |
| MFI sum, median (IQR) | 1911 (1516-12610) | 6967 (848 - 11010) | 0.96 |

Supplementary Table 2: Kidney allograft biopsies histology in each patient

Supplementary Table 3: PBMC populations distribution evolution after IVIG treatment according to induction therapy (thymoglulin – 1 N=4; rIL2 – 2 N=3; no induction – 0 N=4)

| **Populations** | **D0** (**%**.[Q1-Q3]) N=12 | **D30** (**%**. [Q1-Q3]) N=12 | **D30-0** p-value | **D60** (**%**. [Q1-Q3]) N=10 | **D60-0** p-value |
| --- | --- | --- | --- | --- | --- |
| **Monocytes (CD14+)**  **(1)** | **24.7** [13.8 – 37.9] | **29.4** [22.4 – 68.5] | 0.63 | **35.3** [22.8 – 49.9] | 0.50 |
| **(2)** | **21.4** [14.2 – 30.5] | **19.4** [15.7 – 30.8] | 0.88 | **13.9** [12.3 – 17.6] | 0.25 |
| **(0)** | **19.9** [8.6 – 60.5] | **17.1** [7.4 – 46.9] | 0.63 | **7.6** [3.7 – 40.3] | 0.25 |
| **B cells (CD19+)**  **(1)** | **17.9** [11.1 - 20.8] | **11.7** [4.7 - 17.1] | 1.00 | **9.3** [7.8 - 13.7] | 0.25 |
| **(2)** | **3.9** [1.9 - 8.7] | **3.9** [2.0 - 9.7] | 0.15 | **7.8** [4.0 - 9.3] | 0.11 |
| **(0)** | **6.4** [2.4 - 11.8] | **6.6** [3.2 - 10.1] | 0.88 | **6.0** [1.6 - 8.8] | 0.25 |
| **Bm1**  **(1)** | **7.6** [4.4 - 14.5] | **11.1** [5.5 – 14.6] | 0.63 | **13.4** [8.0 – 18.7] | 0.25 |
| **(2)** | **15.8** [11.9 - 19.1] | **15.6** [8.7 – 21.6] | 0.88 | **20.7** [13.6 – 22.6] | 0.25 |
| **(0)** | **14.4** [7.3 - 35.2] | **17.6** [10.3 – 33.0] | 0.55 | **23.8** [12.1 – 42.8] | **0.03** |
| **Bm2**  **(1)** | **73.1** [52.3 – 78.4] | **66.4** [48.4 – 73.3] | 0.25 | **67.8** [37.9 – 67.9] | 0.25 |
| **(2)** | **33** [26.4 – 50.7] | **38.7** [24.6 – 48.9] | 0.88 | **34.7** [18 – 56.9] | 0.75 |
| **(0)** | **37.8** [25.3 – 58.9] | **31.8** [16.2 – 48.9] | 0.38 | **24.6** [14.9 – 47.1] | 0.25 |
| **Bm2’**  **(1)** | **1.9** [0.4 – 7.2] | **3.6** [1.5 – 4.6] | 1.00 | **1.8** [0.7 – 1.9] | 0.75 |
| **(2)** | **0.7** [0.6 – 1.3] | **0.8** [0.6 – 2.8] | 0.25 | **0.5** [0.3 – 0.7] | - |
| **(0)** | **0.9** [0.5 – 2.6] | **1.2** [0.6 – 3.0] | 1.00 | **0.8** [0.5 – 1.6] | 0.64 |
| **Bm5**  **(1)** | **5.5** [3.7 – 15.0] | **7.4** [6.5 – 14.9] | 0.63 | **8.1** [7.9 – 22.8] | 0.25 |
| **(2)** | **22.6** [16.5 – 29.0] | **20.4** [15.2 – 29.7] | 0.88 | **27.8** [8.9 – 32.6] | 1.00 |
| **(0)** | **14.9** [10.2 – 25.1] | **25.4** [16.4 – 31.8] | 0.13 | **18.3** [14.8 – 32.2] | 0.25 |
| **eBm5**  **(1)** | **7.4** [6.1 – 13.0] | **9.4** [8.4 – 17.8] | 0.13 | **11.6** [8.8 – 16.1] | 0.25 |
| **(2)** | **14.1** [7.8 – 20.9] | **18.2** [9.5 – 21.1] | 0.38 | **11.4** [8.4 – 23.3] | 0.75 |
| **(0)** | **9.3** [7.3 – 12.0] | **9.8** [7.9 – 12.4] | 0.88 | **6.3** [5.6 – 10.5] | 0.25 |
| **T cells (CD3+)**  **(1)** | **33.5** [27.8 - 55.0] | **39.6** [18.4 - 55.8] | 1.00 | **44.5** [22.4 - 46.7] | 0.75 |
| **(2)** | **46.7** [32.2 - 60.1] | **48.5** [44.4 - 55.9] | 0.63 | **48.2** [45.5 - 52.8] | 0.25 |
| **(0)** | **58.6** [23.4 - 71.7] | **59.1** [25.3 - 67.3] | 1.00 | **70.1** [26.6 - 79.2] | 0.63 |
| **CD4+**  **(1)** | **41.6** [12.2 - 54.7] | **48.9** [12.2 - 54.7] | 1.00 | **30.1** [26.2 - 52.8] | 1.00 |
| **(2)** | **55.4** [43.5 - 64] | **62.2** [44.9 - 69.9] | 0.25 | **54.3** [40.0 - 55.5] | 1.00 |
| **(0)** | **63.6** [60.3 - 69.9] | **63.9** [51 - 68.1] | 0.50 | **62.3** [52.4 - 72.1] | 0.63 |
| **CD45RA**  **(1)** | **26** [22.9 – 35.6] | **28.4** [20.5 – 42.5] | 1.00 | **26.8** [9.1 – 37.7] | 1.00 |
| **(2)** | **23.4** [16.6 – 42.9] | **22**[20.0 – 39.3] | 0.88 | **18.5** [13.4 – 22.8] | 0.75 |
| **(0)** | **38.4** [8.3 – 55.6] | **21.3** [4.1 – 41.2] | 0.50 | **31.6** [6.8 – 47.7] | 0.13 |
| **CD45RO**  **(1)** | **56.2** [45.2 – 59.6] | **50** [42.5 – 63.2] | 0.75 | **57** [44.2 – 69.5] | 1.00 |
| **(2)** | **48.5** [38.4 – 54.5] | **49.5** [41 – 60.4] | 0.25 | **59.3** [52 – 66.9] | 0.25 |
| **(0)** | **43.3** [30.9 – 76.2] | **59.1** [34.5 – 85.6] | 0.25 | **52.9** [32.3 – 79.9] | 0.25 |
| **Treg**  **(1)** | **11.1** [7.5 – 19.7] | **8.6** [4.4 – 14.9] | 0.38 | **8.4** [6.8 – 10.1] | - |
| **(2)** | **5.1** [2.9 – 15.7] | **5.8** [3.6 – 16.6] | 0.38 | **6.1** [5.1 – 18.9] | 0.75 |
| **(0)** | **4.3** [2.8 – 24.5] | **4.6** [3.5 – 26.5] | 0.38 | **5.3** [3.5 – 25.2] | 0.38 |
| **CD8+**  **(1)** | **33.6** [27.9 - 54.3] | **42.4** [28.8 - 44.1] | 1.00 | **46.2** [38.0 - 63.6] | 0.25 |
| **(2)** | **36.7** [30.2 - 49.2] | **31.1** [26.1 - 47.1] | 0.13 | **40.6** [34.9 - 51.4] | 0.75 |
| **(0)** | **30.4** [25.1 - 34.4] | **27.7** [27.5 - 42.8] | 0.50 | **28.9** [23.1 - 41.5] | 1.00 |
| **CD45RA**  **(1)** | **68.0** [52 – 59.6] | **76.4** [61 – 84] | 0.75 | **59.3** [41.2– 87] | 0.75 |
| **(2)** | **47.5** [25.8 – 54.8] | **45** [37.3 – 51.2] | 1.00 | **37** [16.3 – 50.3] | 0.25 |
| **(0)** | **74.7** [40.1 – 81.2] | **64.5** [31.1 – 68.5] | 0.50 | **70.2** [38.3 – 78.2] | 0.13 |
| **CD45RO**  **(1)** | **8.6** [3.81 –34.5] | **7.6** [2.4 – 10.9] | 0.75 | **12.2** [1.49 – 30] | 0.75 |
| **(2)** | **19.7** [18.1 – 21.5] | **19.5** [17.9 – 22.3] | 1.00 | **27.4** [11.8 – 28] | 0.75 |
| **(0)** | **6.0** [5 – 30.1] | **7.8** [7.25 – 35.3] | 0.75 | **8.64** [7.5 – 34] | 0.13 |
| **NK cells (CD56+)**  **(1)** | **10.8** [6.0 – 21.3] | **9** [2.7 – 10.9] | 0.38 | **11.4** [9.1– 11.8] | 0.50 |
| **(2)** | **24.0** [16.6 – 29.3] | **21.1** [14.3 – 25.5] | 0.38 | **21.7** [18.1– 24.6] | 0.25 |
| **(0)** | **8.2** [4.2 –13.6] | **12.1** [6.3 – 24.6] | 0.13 | **9.0** [6.5– 29.1] | 0.38 |
| **NKT cells (CD3+CD56+)**  **(1)** | **5.0** [0.6– 20.0] | **4.7** [1.9 – 16.9] | 0.88 | **15.4** [2– 25.6] | 0.75 |
| **(2)** | **9.1** [2.4– 22.8] | **7.6** [1.9 – 16.4] | 0.88 | **20.2** [1.6– 22.5] | 1.00 |
| **(0)** | **3.7** [1.0– 15.6] | **5.7** [1.7 – 18.5] | 0.38 | **5.7** [2.3– 9.7] | 0.88 |

Supplementary Table 4: PBMC mRNA transcripts evolution (fold increase) after IVIG treatment according to induction therapy (thymoglulin – 1 N=4; rIL2 – 2 N=3; no induction – 0 N=4)

| **Genes** | **D0** (**Fold increase**.[Q1-Q3]) N=11 | **D30** (**Fold increase**. [Q1-Q3]) N=11 | **D30-0** p-value | **D60** (**Fold increase**. [Q1-Q3]) N=8 | **D60-0** p-value |
| --- | --- | --- | --- | --- | --- |
| **ROR **  **(1)** | 1.00 | **1.02** [0.32 – 4.2] | 0.87 | **4.81** [3.05 – 5.69] | 0.12 |
| **(2)** | 1.00 | **1.13** [0.05 – 3.89] | 0.75 | **1.37** [0.19 – 1.49] | 1.00 |
| **(0)** | 1.00 | **2.54** [0.63 – 3.64] | 0.37 | **1.72** [0.83 – 2.88] | 0.25 |
| **Tbet**  **(1)** | 1.00 | **0.84** [0.23 – 1.28] | 0.62 | **2.54** [1.09 – 4.54] | 0.25 |
| **(2)** | 1.00 | **0.46** [0.18 – 1.01] | 0.50 | **0.61** [0.19 – 0.64] | 0.25 |
| **(0)** | 1.00 | **1.85** [1.08 – 3.63] | 0.25 | **1.4** [0.66 – 13.58] | 0.62 |
| **Gata-3**  **(1)** | 1.00 | **0.33** [0.19 – 0.86] | 0.25 | **0.88** [0.37 – 1.32] | 0.87 |
| **(2)** | 1.00 | **0.52** [0.44 – 0.56] | 0.25 | **1.45** [0.15 – 1.99] | 0.75 |
| **(0)** | 1.00 | **1.04** [0.55 – 2.39] | 0.87 | **1.13** [1.15 – 16.89] | 0.62 |
| **CD3**  **(1)** | 1.00 | **1.20** [0.65 – 2.37] | 0.62 | **3.32** [1.16 – 18.38] | 0.25 |
| **(2)** | 1.00 | **0.81** [0.11 – 3.64] | 1.00 | **2.56** [0.75 – 2.84] | 0.50 |
| **(0)** | 1.00 | **1.78** [0.92 – 3.72] | 0.25 | **1.54** [0.47 – 2.87] | 0.62 |
| **CD32a**  **(1)** | 1.00 | **0.48** [0.42 – 0.88] | 0.12 | **0.78** [0.66 – 1.06] | 0.37 |
| **(2)** | 1.00 | **2.38** [0.58 – 4.53] | 0.50 | **0.97** [0.16 – 3.16] | 1.00 |
| **(0)** | 1.00 | **1.02** [0.49 – 1.89] | 0.75 | **1.15** [0.31 – 2.13] | 0.62 |
| **CD32b**  **(1)** | 1.00 | **0.71** [0.35 – 1.88] | 0.87 | **1.57** [0.88 – 2.25] | 0.37 |
| **(2)** | 1.00 | **0.86** [0.33 – 2.53] | 1.00 | **0.64** [0.48 – 2.87] | 1.00 |
| **(0)** | 1.00 | **2.42** [0.51 – 3.62] | 0.37 | **1.16** [0.51 – 2.72] | 0.62 |
| **CD19**  **(1)** | 1.00 | **0.93** [0.65 – 1.43] | 1.00 | **3.41** [2.01 – 6.68] | 0.12 |
| **(2)** | 1.00 | **0.51** [0.08 – 0.71] | 0.25 | **0.9** [0.72 – 1.77] | 1.00 |
| **(0)** | 1.00 | **1.78** [0.92 – 5.87] | 0.25 | **0.78** [0.38 – 1.73] | 1.00 |
| **BAFF**  **(1)** | 1.00 | **1.09** [0.70 – 1.30] | 1.00 | **1.45** [0.59 – 1.67] | 0.62 |
| **(2)** | 1.00 | **4.26** [1.56 – 13.93] | 0.25 | **1.99** [0.83 – 3.61] | 0.50 |
| **(0)** | 1.00 | **1.17** [0.89 – 2.22] | 0.37 | **1.85** [0.5 – 6.34] | 0.62 |
| **BAFF-R**  **(1)** | 1.00 | **0.87** [0.22 – 3.54] | 1.00 | **1.9** [1.39 – 2.17] | 0.12 |
| **(2)** | 1.00 | **1.23** [0.13 – 1.43] | 1.00 | **0.81** [0.03 – 0.82] | 0.25 |
| **(0)** | 1.00 | **2.32** [0.84 – 4.06] | 0.25 | **1.63** [0.41 – 5.07] | 0.62 |
| **TGFβ**  **(1)** | 1.00 | **1.34** [0.30 – 2.29] | 0.62 | **0.54** [0.43 – 0.6] | 0.12 |
| **(2)** | 1.00 | **2.73** [0.3 – 8.82] | 0.50 | **0.45** [0.21 – 13.59] | 1.00 |
| **(0)** | 1.00 | **1.77** [0.89 – 2.06] | 0.25 | **1.50** [1.26 – 5.95] | 0.12 |
| **Fas**  **(1)** | 1.00 | **0.98** [0.32 – 3.02] | 1.00 | **0.87** [0.70 – 1.69] | 1.00 |
| **(2)** | 1.00 | **3.33** [0.28 – 3.68] | 0.50 | **0.19** [0.18 – 1.21] | 0.50 |
| **(0)** | 1.00 | **0.54** [0.32 – 0.93] | 0.25 | **0.75** [0.05 – 1.67] | 0.62 |
| **FasL**  **(1)** | 1.00 | **0.93** [0.26 – 1.12] | 0.62 | **2.35** [1.11 – 4.44] | 0.25 |
| **(2)** | 1.00 | **0.88** [0.07 – 0.98] | 0.25 | **0.63** [0.62 – 0.66] | 0.25 |
| **(0)** | 1.00 | **3.18** [0.38 – 6.29] | 0.62 | **3.67** [0.84 – 6.34] | 0.25 |
| **CD4**  **(1)** | 1.00 | **1.85** [0.72 – 2.15] | 0.25 | **1.58** [0.97 – 2.37] | 0.25 |
| **(2)** | 1.00 | **0.99** [0.35 – 2.47] | 1.00 | **1.57** [0.71 – 1.64] | 0.50 |
| **(0)** | 1.00 | **0.89** [0.32 – 1.97] | 1.00 | **1.06** [0.5 – 7.30] | 0.87 |
